# Can Treadmill Perturbations Evoke Stretch Reflexes in the Calf Muscles?

**DOI:** 10.1371/journal.pone.0144815

**Published:** 2015-12-15

**Authors:** Lizeth H. Sloot, Josien C. van den Noort, Marjolein M. van der Krogt, Sjoerd M. Bruijn, Jaap Harlaar

**Affiliations:** 1 Dept. of Rehabilitation Medicine, MOVE Research Institute Amsterdam, VU University Medical Center, Amsterdam, the Netherlands; 2 MOVE Research Institute Amsterdam, Faculty of Human Movement Sciences, VU University, Amsterdam, the Netherlands; 3 Department of Orthopedics, First Affiliated Hospital of Fujian Medical University, Fuzhou, Fujian, People's Republic of China; Semmelweis University, HUNGARY

## Abstract

Disinhibition of reflexes is a problem amongst spastic patients, for it limits a smooth and efficient execution of motor functions during gait. Treadmill belt accelerations may potentially be used to measure reflexes during walking, i.e. by dorsal flexing the ankle and stretching the calf muscles, while decelerations show the modulation of reflexes during a reduction of sensory feedback. The aim of the current study was to examine if belt accelerations and decelerations of different intensities applied during the stance phase of treadmill walking can evoke reflexes in the gastrocnemius, soleus and tibialis anterior in healthy subjects. Muscle electromyography and joint kinematics were measured in 10 subjects. To determine whether stretch reflexes occurred, we assessed modelled musculo-tendon length and stretch velocity, the amount of muscle activity, as well as the incidence of bursts or depressions in muscle activity with their time delays, and co-contraction between agonist and antagonist muscle. Although the effect on the ankle angle was small with 2.8±1.0°, the perturbations caused clear changes in muscle length and stretch velocity relative to unperturbed walking. Stretched muscles showed an increasing incidence of bursts in muscle activity, which occurred after a reasonable electrophysiological time delay (163–191 ms). Their amplitude was related to the muscle stretch velocity and not related to co-contraction of the antagonist muscle. These effects increased with perturbation intensity. Shortened muscles showed opposite effects, with a depression in muscle activity of the calf muscles. The perturbations only slightly affected the spatio-temporal parameters, indicating that normal walking was retained. Thus, our findings showed that treadmill perturbations can evoke reflexes in the calf muscles and tibialis anterior. This comprehensive study could form the basis for clinical implementation of treadmill perturbations to functionally measure reflexes during treadmill-based clinical gait analysis.

## Introduction

Spasticity is one of several problems regularly faced by patients with cerebral palsy, spinal cord injury, stroke or multiple sclerosis. It is traditionally defined as a velocity-dependent increase in muscle tone due to hyper excitability of the stretch reflexes [1]. Different treatments are available to reduce the effects of spasticity, such as chemical denervation of muscles by botulinum toxin injections to weaken the muscles or reduction of dorsal nerve roots by rizhotomy to reduce sensory afferents. To select a treatment for a patient, clinicians typically rely on unloaded and passive stretch measurements based on imposed movement of one joint, while asking the patient to relax. However, stretch reflexes should preferably be assessed during the affected common daily activities, such as gait, especially since the actual contribution of exaggerated reflexes to gait deviations has become subject of debate [2;3]. Therefore, it would be much more meaningful to be able to measure reflex activity during gait.

Different approaches have been used to evoke stretch reflexes of the lower leg muscles during gait, ranging from hammer tests to tap on the tendon [4], electromechanical tendon vibrations [5], electrical stimulation of the tibial nerve [6;7], as well as functional [8–10] and other electromechanical perturbations [11–15]. Out of these methods, the tendon tapping, vibration and nerve stimulation are not practical and uncomfortable to use during patient measurements. The functional perturbations are more clinically feasibly, because they use different walking speeds to lengthen the muscles at different stretch velocities. The relation between muscle activity and muscle stretch velocity is than used as an indication of the strength of the stretch reflex [16]. Using this method, exaggerated muscle responses were found for the calf muscles during the swing phase in spastic patients compared with healthy subjects [8;17]. However, during the stance phase, in which the calf muscles are active, it is more complicated to discern the contribution of reflexes from central driven muscle activation.

Electromechanical perturbations of the muscle have been applied by several groups using actuated joint orthoses. These orthoses have been used to suddenly lift the forefoot or directly rotate the ankle towards dorsiflexion to stretch the calf muscles during different phases of gait [12–14]. In the soleus of healthy adults and children, both mono- and polysynaptic responses were measured [12–14;18–20], and found to be modulated during the gait cycle [18;21]. In different groups of spastic patients, the monosynaptic response was found to be exaggerated while its modulation was hampered [20–22]. To examine the actual contribution of the stretch reflex circuitry to muscle activity, plantar flexion rotations were also applied. The induced shortening of the calf muscles caused a decrease in the length and load sensitive feedback, which was followed by a drop in soleus activity [14;19;23]. Similar reductions were found for control children and children with cerebral palsy [20], while the activity only slightly reduced in stroke patients [22;24]. Although these actuated joints allow for reflex assessment during gait, they can also interfere with gait performance by their mass, straps and restriction in medio-lateral movement [12–14;24]. A less obtrusive approach is to apply ankle rotations using an actuated platform that rotates in the sagittal plane [15]. However, the applicability is limited for it is rather cumbersome to obtain sufficient steps with correct alignment between the ankle axis and the rotation platform.

Alternatively, electromechanical perturbations have been applied during the eighties by acceleration impulses of the belt while subjects were walking on a treadmill [11]. Such an acceleration pulls the foot backward, thereby causing a quick dorsiflexion of the ankle and stretching of the calf muscles. On the spastic side of hemiparetic patients, these perturbations resulted in large short-latency responses, i.e. a monosynaptic stretch reflexes through stimulation of the group Ia muscle spindles [25]. On their unaffected side, and in healthy subjects, only long-latency responses were measured, representing polysynaptic spinal reflex activation through group II muscle spindles [11;25]. Although these results seem promising, the treadmill accelerations have not been further used to evoke reflexes in the calf muscles, nor has this approach been clinically implemented to date.

With the introduction of instrumented treadmills in rehabilitation centers, it becomes more and more feasible to incorporate the acceleration-based treadmill perturbations to functionally measure reflexes during treadmill-based clinical gait analysis. However, a more comprehensive study of the responses of the lower leg muscles to such perturbations is necessary. First, because the original studies focused only on the gastrocnemius, while it is important for treatment planning to distinguish between the gastrocnemius and soleus muscle and to include the behavior of the antagonist muscle. In addition, a more quantitative description of the muscle response is needed, including musculoskeletal modeling of change in muscle length and velocity thereof, with multiple perturbation intensities to calculate the muscle response strength. The use of deceleration perturbations allows for examination of stretch of the tibialis anterior and the effect of a reduction of length and velocity feedback in the calf muscles. Finally, performing such experiments in healthy persons would provide normative data to evaluate potential pathological responses in patients against using the current technology and equipment.

Therefore, the aim of the current study was to examine whether different treadmill perturbations applied to the ankle during the stance phase of walking can evoke stretch reflexes in healthy gastrocnemius (medialis and lateralis), soleus and tibialis anterior muscles using belt acceleration and deceleration of different intensities.

## Methods

Ten healthy subjects (age: 24.8 ± 2.0 yr; BMI: 23.0 ± 2.0 kg/m^2^; 5 female) were included in the study. They did not have former surgery or current injuries to the lower extremities. Subjects gave written informed consent. The study was approved by the Institutional Review Board of the Faculty of Human Movement Sciences, VU University, Amsterdam, The Netherlands.

Subjects walked at a fixed speed of 1.2 ms^-1^ on a split-belt instrumented treadmill (GRAIL, Motekforce Link, the Netherlands). They wore comfortable flat-soled shoes and a safety harness during the experiment. First, subjects were given five minutes to familiarize to the set-up and the perturbations. Then, several trials of three minutes were performed, during which acceleration (ACC) or deceleration (DEC) perturbations of belt speed were applied. Both type of perturbations consisted of 5 different intensities, set to reach maximum velocity differences of 0.1 to 0.5 with increments of 0.1 ms^-1^. Five different intensities were applied to explore the relationship between intensity and their effect. Fifteen repetitions of each intensity were randomly applied within a window of 10 strides, with a recovery period of at least 5 strides, which appeared to be sufficient time to recover to a normal gait pattern (see S1 Fig). The perturbations were only applied to the right leg. They were triggered by heel strike, which was based on heel and sacrum marker data [26], around 10 to 15% of the gait cycle. The perturbations occurred during stance phase and ended before 50% of the gait cycle.

Ground reaction forces and moments were measured by the force sensors mounted underneath both treadmill belts (50x200 cm) and belt speed was registered by the treadmill’s controller, both at 1000 Hz. Motion data were captured at 100 Hz via a passive motion capture system (Vicon, Oxford, UK) EMG electrodes (Ø 15mm, 24mm inter-electrode distance) were attached on the m. gastrocnemius medialis (GM) and lateralis (GL), m. soleus (SO) and m. tibialis anterior (TA) according to the SENIAM guidelines [27]. EMG was measured at 1000 Hz via a wireless system (Wave EMG system, Cometa, Italy).

### Data processing

3D joint angles for hip, knee and ankle were calculated following the CAMARC anatomical frame definitions [28], using the open source Matlab software (BodyMech). The following bony landmarks were used as input: the anterior and posterior superior iliac spines for the pelvis, trochanter major, epicondylus lateralis and medialis for the thigh, caput fibulae, tuberositas tibiae, malleolus lateralis and medialis for the shank and the calcaneus and caput metatarsale I and V for the foot. Musculo-tendon lengths (MTL) of the GM, GL, SO and TA were calculated using a generic gait model (2392) in musculoskeletal modeling software (OpenSim) [29]. The generic model was scaled to fit the individual subject’s size and matched to the subject’s kinematics using the inverse kinematics tool in OpenSim.

**Fig 1:**
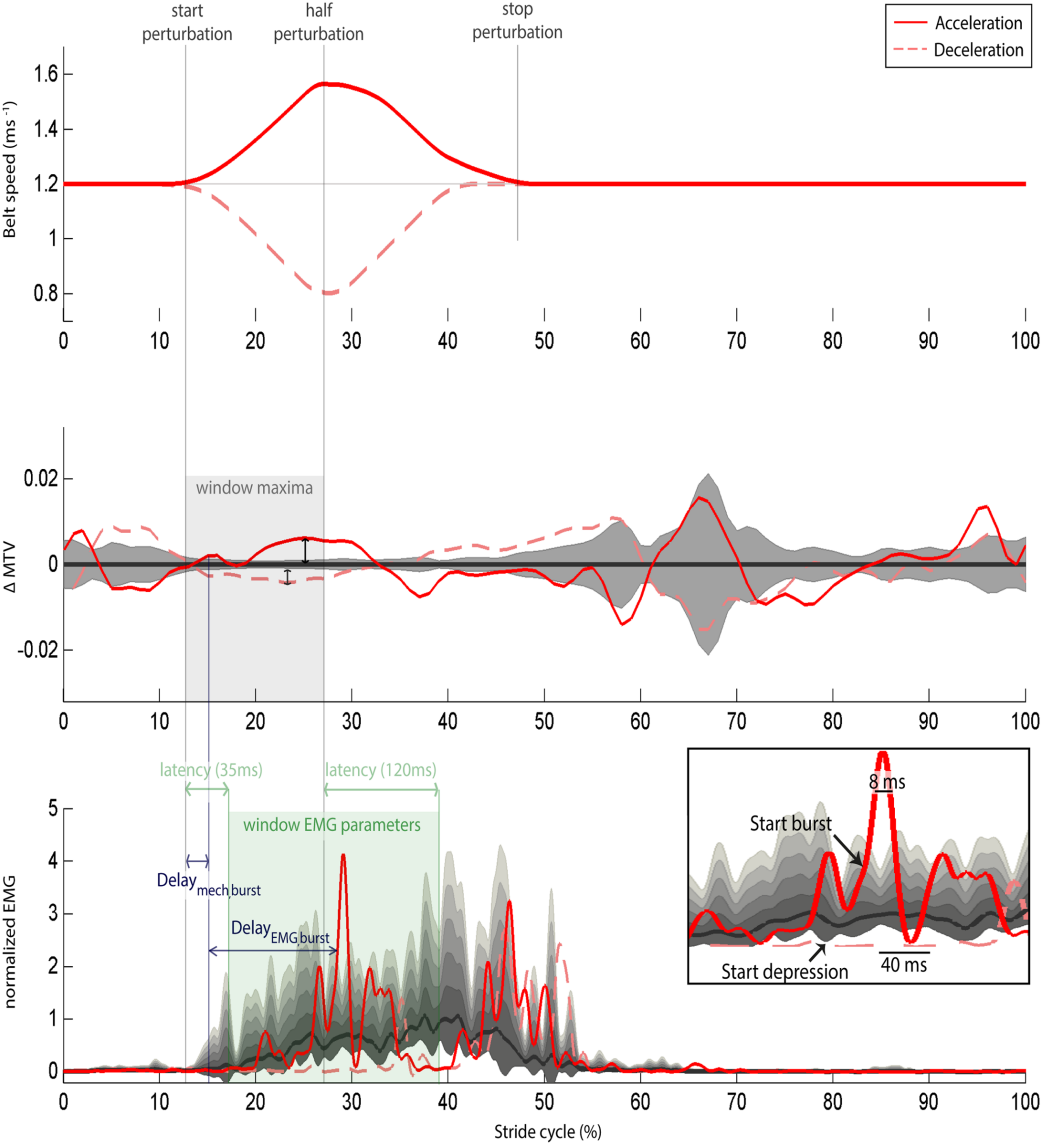
Parameter definitions. To illustrate the definitions of the different parameters, the unperturbed data of one subject is shown, with the mean as solid line and one standard deviation indicated by the gray area, as well as one perturbation of the highest intensity for both ACC (red solid line) and DEC (red striped line). The start, half, and stop of the perturbation (indicated for ACC by vertical grey lines) are derived from the belt speed, with half perturbation the maximum (ACC) or minimum (DEC) belt speed. Within the window (gray)_of start to half perturbation, the maximum deviation of MTV from the average unperturbed strides (ΔMTV) is calculated. The maximum deviation from unperturbed knee angle, ankle angle and MTL are also calculated within this window (not shown). The lowest graph shows the EMG normalized to the maximum of the ensemble averaged unperturbed strides. For each signal, it is determined if there was a burst and a depression in the EMG within the window indicated as ‘window EMG parameters’ (in green). This is from start perturbation plus 35 ms (i.e. theoretical minimum reflex latency) until half perturbation plus 120 ms (i.e. theoretical maximum reflex latency). An EMG burst is defined as a signal above the burst threshold, i.e. five standard deviation of unperturbed EMG, for a minimum duration of 8 ms (see inset). A depression in EMG is defined as signal lower than the depression threshold, i.e. one standard deviation of unperturbed EMG, for a minimum duration of 40 ms (see inset). In case of a burst or depression, the mechanical delay and electrophysiological delay are calculated. The mechanical delay (delay_mech,burst_ or delay_mech,depres_) describes the delay from start perturbation until a change in MTV that could trigger a stretch reflex, in this study conservatively defined as one standard deviation of the unperturbed strides (indicated in blue). The electrophysiological delay of a burst (delay_EMG,burst_) is defined as the time from the mechanical delay until the moment of exceeding the burst threshold (indicated). Similarly, the electrophysiological delay of a depression (delay_EMG,depr_) is the time from the mechanical delay until the signal exceeds the depression threshold (not indicated).

MTL was modeled according to the muscle attachment sites and moment arms around the joints. Muscle-tendon stretch velocity (MTV) was obtained by differentiating MTL followed by low-pass filtering (symmetrical 4^th^ order Butterworth filter at 20 Hz). MTL and MTV were non-dimensionalized by dividing MTL by the anatomical reference length with all joint angles set at zero (*l*_ref_) and MTV by dividing by $\sqrt{g}\times l_{ref}$ [8;30]. EMG was high-pass filtered at 20Hz to remove movement artifacts, rectified, low-pass filtered at 50Hz (both with a symmetrical 4^th^ order Butterworth filter) and normalized to the maximum of the ensemble averaged unperturbed strides of the specific muscle. In contrast to the perturbation control, heel strikes and toe-offs were detected from the force plate data during offline analysis and used to time-normalize all data based on spline interpolation [31]. Start and end of the perturbations were determined from the derivative of the belt speed. Only the effect of the first half of the perturbations was examined, i.e. the acceleration phase for ACC and deceleration phase of DEC (Fig 1), and lasted from start of the perturbation to the moment of maximum belt speed difference.

Since there is no generally accepted parameter to represent stretch reflexes, we formulated the following conditions: 1) the perturbation should result in a change in MTL and MTV relative to unperturbed walking, increasing with perturbation intensity (mechanical response); 2) after exceeding the mechanical threshold (i.e. muscle stretch velocity), a burst of muscle activity should appear in the stretched muscle (electrophysiological response); 3) this burst should occur after some expected time delay; 4) the amplitude of the response should increase with increasing muscle stretch velocity; and 5) the increase in EMG should not represent co-contraction as demonstrated by simultaneous activation of the antagonist muscle(s).

To verify condition 1, the maximum positive (ACC) or negative (DEC) deviation from the average of the unperturbed trials were calculated for the sagittal ankle and knee angle, as well as MTL and MTV for the different muscles.

To test condition 2, we calculated the average deviation (root-mean-square, RMS) in EMG from unperturbed walking. In addition, we determined the percentage of trials with a burst and the percentage of trials with a depression. An increase, or burst, in EMG was defined as an exceeding of five standard deviations (std) of the unperturbed strides for at least 8 ms to exclude outliers [32]. A depression in EMG was defined as a decrease in EMG of more than one std for at least 40 ms, because a reduction in afferent feedback in the lengthened muscle is expected to result in a slight decrease in muscle activity over a longer period [33]. These parameters were determined within the time window from start of the perturbation plus the shortest reflex delay (35 ms) to the end of the stretch phase plus the longest long reflex delay (120 ms; Fig 1), with the delay values based on literature [18].

To verify condition 3, for each burst and depression the mechanical (delay_mech,burst_ and delay_mech,depres_) and electrophysiological (delay_EMG,burst_ and delay_EMG,depres_) time delays were determined. The delay_mech_ was defined as the time between perturbation onset and a change in MTV larger than one std of the unperturbed strides, assuming that the stretch reflexes were primarily velocity-dependent. The delay_EMG_ was defined as the time between delay_mech_ and EMG onset, i.e. the instant of exceeding 5 std (delay_EMG,burst_) or 1std (delay_EMG,depres_). Time delays were reported if both delay_EMG_ and delay_mech_ were found and if sufficient data were present, i.e. EMG onset in at least 20% of the trials per condition, with an average of at least 2 values per intensity for a subject.

To test condition 4, the strength of the general muscle response was quantified as the ratio between peak delta EMG and peak increase (ACC) or decrease (DEC) in delta MTV. In addition, the correlation of specifically the burst amplitude to maximum stretch velocity was examined for the trials with a burst to examine the underlying assumption of the relation between muscle activity and stretch velocity.

To verify condition 5, the average co-contraction index (CCI) was calculated by dividing the absolute difference in EMG by the sum of the EMG, according to:

$$CCI=1-\frac{\left|EMG_{ag}-EMG_{ant}\right|}{EMG_{ag}+EMG_{ant}}$$

with EMG_ag_ the muscle activity of the agonist muscle and EMG_ant_ of the antagonist muscle for each moment within the previous described time window, and subsequently averaged [34]. A value of one represents pure co-contraction and zero an absence of simultaneous muscle activity.

Finally, the general effect on the gait pattern was examined using spatio-temporal parameters, including stride length, stride time, stance phase and step width. The previous described analyses were also performed on the unperturbed strides for comparison. Unperturbed strides were selected as the stride that preceded the perturbed strides. All parameters described above were calculated using the average onset and ending of the perturbations.

### Statistics

We tested, using an ANOVA for repeated measures, all the parameters described above, i.e. deviation in ankle and knee angle, MTL and MTV, RMS EMG, % bursts, % depressions, mechanical and electrophysiological time delays of bursts and depressions, CCI, strength of muscle response, stride length, stride time, stance phase and step width for the 4 different muscles (if applicable) and for ACC and DEC. First, we examined if there was an effect of the perturbations, i.e. if the parameters differed between unperturbed and perturbed walking, using a Helmert contrast. Secondly, we examined if there was an effect of intensity on these parameters, by comparing the different intensities. The effect of intensity was also examined for parameters describing the perturbations, including difference in belt speed and acceleration as well as in ankle angular velocity, time to maximum belt speed and start and duration of the perturbation. P<0.05 was considered significant and Greenhouse-Geisser correction was used in case of violations of the sphericity assumption. If significant, Tukey LSD post-hoc tests were performed, with p<0.01 as significant thereby correcting for multiple testing per parameter.

To test the correlation between burst amplitude and muscle stretch velocity (condition 4), linear regression analysis was performed per muscle using generalized estimating equations (GEE), with burst amplitude as dependent and maximum MTV as covariate (exchangeable working correlation structure and with a robust estimation. Calculations and statistical analyses were performed in Matlab (The Mathworks Inc., Natick MA) and IBM SPSS Statistics (Armonk, NY, USA).

## Results

### Perturbations

**Fig 2:**
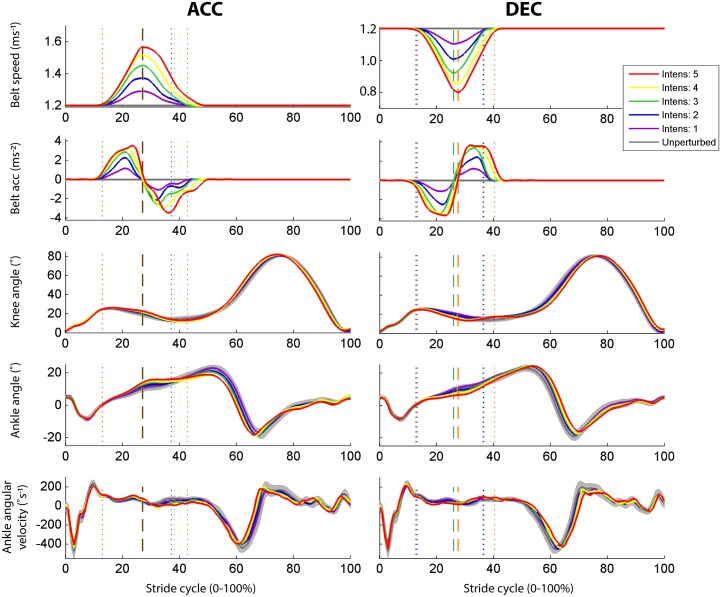
Perturbation characteristics. Details of the different intensities of ACC (left) and DEC (right). Effects are shown for belt speed and acceleration, knee and ankle angle and ankle angular velocity for ACC for a typical subject (same subject is selected for all exemplary figures). Start and end of the perturbation (dotted lines) and half of the perturbations (i.e. maximum velocity; dashed line) are indicated. The standard deviation of the unperturbed strides is shown in gray.

Since the measured muscle response is dependent on the perturbation, the characteristics of the perturbations are first precisely described in Fig 2 and Table 1. In line with the imposed settings, the difference in belt speed increased with intensity for both ACC and DEC (p<0.001), although less than the 0.1 ms^-1^. The maximum acceleration remained similar after the second intensity, so time to maximum velocity change and perturbation duration increased slightly for the higher intensities (p<0.001). Both perturbation types started on average 147±22 ms after heel strike, and this timing did not differ between intensities (p = 0.49). Maximum ankle angular velocity increased with perturbation intensity (p<0.001).

**Table 1:** Perturbation characteristics.

	Intensity 1	Intensity 2	Intensity 3	Intensity 4	Intensity 5	Effect intensity
**Acceleration**						
Max. delta speed (ms^-1^)	0.09±0.01^2−5^	0.19±0.00^3−5^	0.28±0.00^4−5^	0.36±0.01^5^	0.42±0.01	**0.000**
Max. delta acc. (ms^-2^)	0.17±0.03^2−5^	0.28±0.00^-^	0.29±0.00^-^	0.28±0.00^-^	0.29±0.00	**0.000**
Time to max. velocity (ms)	137±12^3−5^	138±12^3−5^	149±10^4,5^	166±10^5^	179±10	**0.000**
Duration perturbation (ms)	278±25^3−5^	278±25^3−5^	290±23^4,5^	314±22^5^	337±18	**0.000**
Start perturbation (ms)	146±23	144±16	145±17	148±19	146±16	0.490
Max. delta angular vel. (°s^-1^)	32.82±5.42^5^	34.29±5.45^4,5^	38.58±7.13^5^	44.02±10.20^-^	45.95±8.99	**0.000**
**Deceleration**						
Min. delta speed (ms^-1^)	-0.09±0.01^2−5^	-0.19±0.00^3−5^	-0.28±0.00^4−5^	-0.35±0.01^5^	-0.42±0.02	**0.000**
Min. delta acc. (ms^-2^)	-0.17±0.03^2−5^	-0.28±0.00^-^	-0.28±0.00^-^	-0.28±0.01^-^	-0.29±0.01	**0.000**
Time to max. velocity (ms)	138±13^3−5^	139±13^3−5^	149±12^4,5^	165±9^5^	180±11	**0.000**
Duration perturbation(ms)	277±26^3−5^	278±26^3−5^	291±24^4,5^	312±21^5^	339±20	**0.000**
Start perturbation (ms)	149±32	146±22	144±22	144±25	147±27	0.586
Min. delta Angular vel. (°s^-1^)	-34.15±8.77^2−5^	-36.65±5.43^5^	-40.29±6.67^5^	-46.75±8.64^-^	-50.13±7.61	**0.000**

with max. as maximum, min. as minimum, acc. as acceleration and vel. as velocity. Grand means and standard deviations are given. Results of the post-hoc tests are indicated with in superscript the following intensities a trial differs from (p<0.01).

### Effect of ACC

ACC pulled the foot backwards and caused both the ankle and knee to flex relative to the unperturbed strides (both p<0.002), which increased with intensity up to 2.8±1.0 and 2.2±0.9 °, respectively (both p<0.001; Fig 2). In turn, this flexion caused an elongation of the calf muscles (GM, GL and SO) and shortening of TA compared with unperturbed strides (all p<0.001). The peak muscle length and stretch velocity changes increased with intensity (all p<0.002).

**Fig 3:**
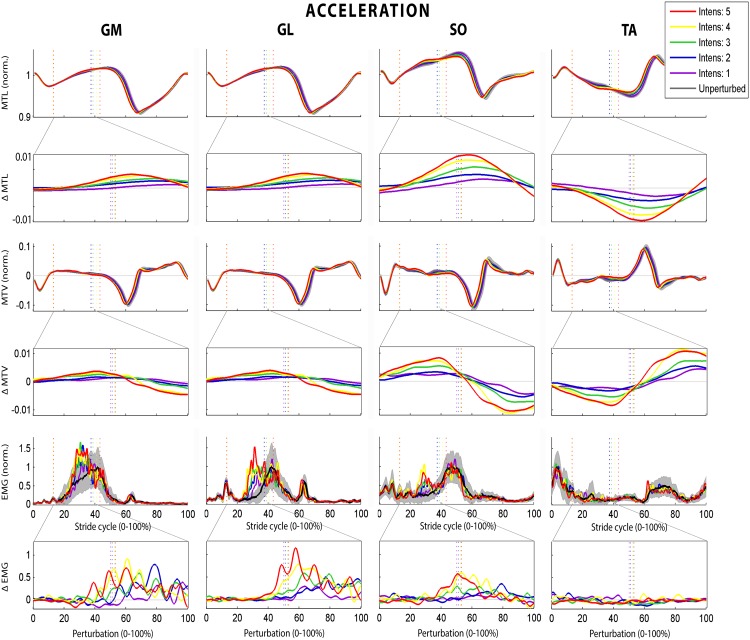
Effect of the different ACC intensities on the different muscles for a typical subject. For the muscle-tendon length (MTL), length change rate and normalized EMG a stride-normalized plot (with the start and end of the perturbations indicated by the dotted lines and unperturbed standard deviation in gray) is shown as well as a perturbation-normalized plot of the difference (Δ) with the unperturbed strides (with half of the perturbations indicated by the dotted line).

An example of the muscle response to ACC perturbations is given in Fig 3. Similar patterns with a clear response in the calf muscles were observed for most subjects (GM: 9/10 subjects; GL and SO: 7/10). The amount of EMG as well as the incidence of bursts of EMG of the calf muscles increased compared with unperturbed strides (all p<0.002) and both increased with intensity (all p<0.002), while there were no differences for TA. Both the mechanical and EMG delay of these bursts did not change with intensity, with delay_mech_ ranging from 28 to 53 ms and delay_EMG_ from 163 to 191 ms. ACC did not result in depression of muscle activity of the calf muscles nor of TA. Response strength (relation between muscle response and MTV) increased with intensity for GM and GL (*p*≤0.001) and showed a similar trend for SO (*p* = 0.08), while there was no effect for TA. Peak bursts was positively related to MTV for all calf muscles (all *p*≤0.003). The level of co-contraction of the calf muscles with TA was similar for GM and even higher for GL and SO during unperturbed compared with perturbed walking (both *p*≤*0*.*001*). Co-contraction did not change with intensity for GM and SO, and decreased with intensity for GL (*p =* 0.004).

**Fig 4:**
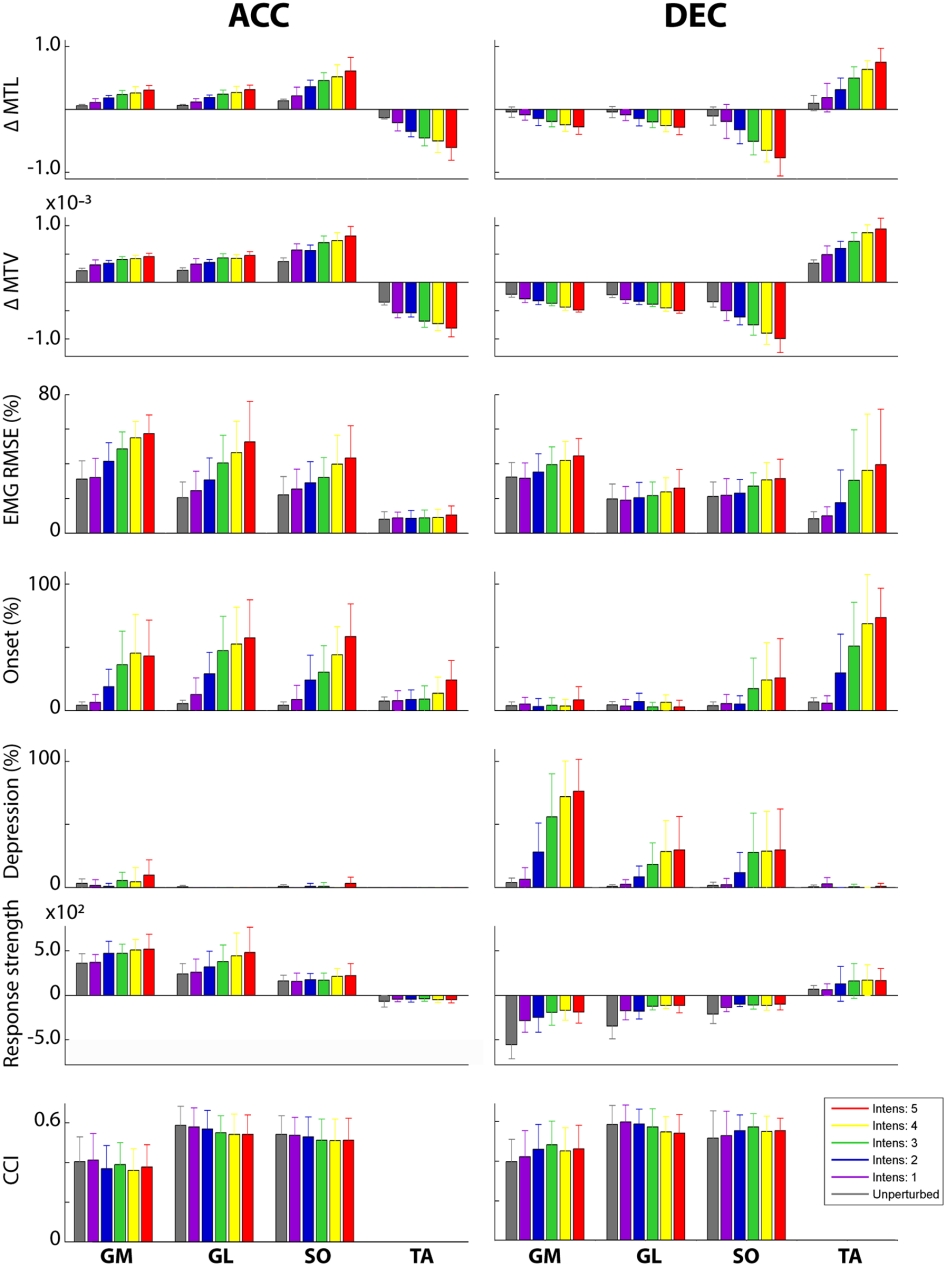
The effect of ACC and DEC and the different intensities (I). Mean and standard deviation is shown, with unp. As unperturbed and CCI as the co-contraction index quantifying the co-contraction of the calf muscles relative to the TA muscle.

The perturbations slightly affected the gait pattern, with a decreased stance phase duration and stride time as well as increased stride length (all p<0.003). These effects increased with intensity (all p<0.003), while step width was not affected. An overview of the effects of ACC is given in Fig 4 and Table 2.

**Table 2:** Parameters ACC.

Parameters	UNPERTURBED		PERTURBED					
		Effect P	I1	I2	I3	I4	I5	Effect I
**Kinematics**								
Max angle (°)								
ankle	0.6±0.1^2−5^	**0.000**	1.1±0.3^3−5^	1.6±0.5^3−5^	2.0±0.4^-^	2.3±0.8^-^	2.8±1.0	**0.000**
knee	0.8±0.2^2−5^	**0.001**	0.9±0.9^4−5^	1.4±0.5^-^	1.7±0.7^-^	2.0±0.8^-^	2.2±0.9	**0.000**
**Muscle**								
Max MTL (x10^-3^)								
GM	0.6±0.2^2−5^	**0.000**	1.1±0.6^2−5^	1.8±0.4^5^	2.4±0.6^5^	2.6±1.0^-^	3.1±0.7	**0.001**
GL	0.6±0.2^2−5^	**0.000**	1.2±0.6^2−5^	1.9±0.4^5^	2.5±0.6^5^	2.7±1.0^-^	3.2±0.8	**0.001**
SO	1.4±0.3^2−5^	**0.000**	2.2±1.4^3−5^	3.6±1.0^4,5^	4.6±1.3^-^	5.1±1.9^-^	6.1±2.2	**0.000**
Min MTL (x10^-3^)								
TA	-1.3±0.2^2−5^	**0.000**	-2.1±1.3^3−5^	-3.5±1.0^4,5^	-4.5±1.2^-^	-5.1±1.9^-^	-6.0±2.1	**0.000**
Max MTV (x10^-3^)								
GM	2.8±0.4^1−5^	**0.000**	3.1±0.8^3,5^	3.3±0.6^3,5^	4.0±0.5^-^	4.2±0.6^-^	4.5±0.6	**0.000**
GL	2.1±0.4^1−5^	**0.000**	3.2±1.0^3,5^	3.5±0.5^3,5^	4.3±0.8^-^	4.3±0.6^-^	4.8±0.6	**0.000**
SO	3.7±0.7^1−5^	**0.000**	5.7±1.1^3−5^	5.6±1.0^3−5^	7.0±1.2^-^	7.4±1.4^-^	8.2±1.7	**0.000**
Min MTV (x10^-3^)								
TA	-3.5±0.6^1−5^	**0.000**	-5.1±1.8^3−5^	-5.4±0.7^3−5^	-6.8±1.1^-^	-7.2±1.2^-^	-8.1±1.5	**0.000**
**EMG**								
RMS (%)								
GM	31±11^2−5^	**0.000**	32±11^2−5^	41±11^4,5^	49±10^4,5^	55±10^-^	58±11	**0.000**
GL	20±9^2−5^	**0.001**	24±11^3−5^	31±13^3−5^	40±16^5^	46±18^-^	53±24	**0.000**
SO	22±10^1−5^	**0.001**	25±11^3−5^	29±12^4,5^	32±12^5^	40±17^-^	43±18	**0.000**
TA	8±4	0.180	9±3	8±4	9±5	9±5	10±5	0.289
Bursts (%)								
GM	2±2^2−5^	**0.001**	4±5^3−5^	15±12^3,5^	36±27^-^	42±31^-^	43±28	**0.000**
GL	4±2^2−5^	**0.000**	12±14^3−5^	27±17^3,5^	47±28^-^	52±29^-^	57±30	**0.000**
SO	2±2^3−5^	**0.000**	7±10^3−5^	19±20^4,5^	28±20^4,5^	42±24^-^	58±26	**0.000**
TA	5±3	0.404	6±6^-^	4±5^-^	3±6^5^	6±8^5^	15±12	0.034
Delay_mech,burst_ (ms)								
GM					28±18	35±24	42±26	0.332
GL				53±41	33±18	36±17	46±22	0.158
SO					36±25	31±20	33±21	0.802
Delay_EMG,burst_ (ms)								
GM					179±38	182±25	188±33	0.885
GL				163±43	173±20	173±24	167±22	0.881
SO					166±25	191±32	178±36	0.189
Depression (%)								
GM	2±3	0.189	1±5	1±2	5±7	4±11	9±13	0.185
GL	0±1	0.343	0±0	0±0	0±0	0±0	0±0	1.000
SO	0±1	0.156	0±0	1±2	1±3	0±0	3±5	0.161
TA	0±0	1.000	0±0	0±0	0±0	0±0	0±0	1.000
Strength (x10^3^)								
GM	36±11^-^	**0.046**	37±15^2−5^	51±14^-^	50±12^-^	53±14^-^	54±15	**0.005**
GL	24±13^3−5^	**0.011**	26±15^3−5^	32±17^5^	38±19^-^	44±25^-^	48±28	**0.001**
SO	16±7	0.071	16±9	17±7	17±8	21±9	22±1	0.080
TA	-7±7	0.260	-4±3	-0±3	-4±3	-5±3	-5±4	0.318
CCI (%)								
GM	39±13	0.437	41±13^-^	37±12^-^	39±11^-^	36±11^-^	38±11	0.040
GL	59±9^3−5^	**0.000**	58±10^4,5^	57±9^5^	55±09^-^	54±10^-^	54±10	**0.004**
SO	54±9^3,4^	**0.001**	54±9	53±10	51±11	51±11	51±11	0.175
**Spatiotemporal**								
Stride length (m)	1.32±0.04^1−5^	**0.000**	1.34±0.05^3−5^	1.35±0.05^5^	1.35±0.04^5^	1.35±0.04^5^	1.37±0.04	**0.000**
Stride time (s)	1.10±0.04^4,5^	**0.002**	1.10±0.04^4,5^	1.10±0.04^4,5^	1.09±0.03^4,5^	1.08±0.03^-^	1.08±0.03	**0.000**
Stance phase (%)	66.9±0.7^1−5^	**0.000**	65.5±0.8^2,3,5^	65.1±0.8^-^	64.9±0.8^-^	64.9±0.9^-^	64.6±1.0	**0.002**
Step width (cm)	10.4±2.6^-^	0.243	10.4±2.8^-^	10.2±2.5^-^	10.2±2.3^-^	10.3±2.3^-^	10.2±2.3	0.768

With max maximum, min minimum, RMS root-mean-square, CCI co-contraction index, Strength the response strength and I Intensity. Grand means and standard deviations are given. Mechanical and EMG delays are only shown if sufficient group data was available. Effect P shows the p-values of the significant difference between unperturbed versus all perturbed and Effect I shows the p-values of the effect of intensity. Results of the post-hoc tests are indicated with in superscript the higher intensities that differ from a specific trial (p<0.01).

### Effect of DEC

Similar but opposite effects were found for DEC compared with ACC. DEC resulted in an increasing ankle plantarflexion and knee extension (p<0.005; Fig 2). Calf muscle length decreased with intensity (all p<0.001) and TA length increased (p<0.001), all with increasing muscle stretch velocity (all p<0.001).

**Fig 5:**
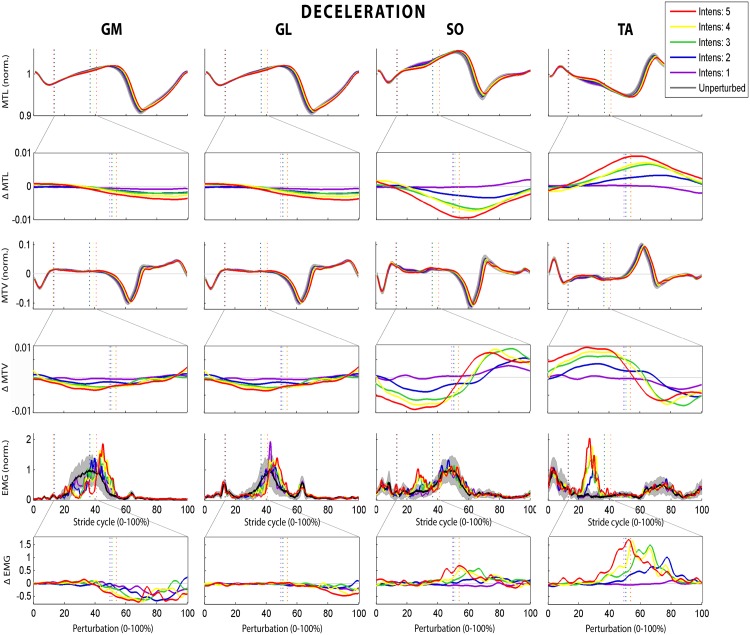
The effect of the different DEC intensities on the different muscles for a typical subject. For the muscle-tendon length (MTL), length change rate and normalized EMG a stride-normalized plot (with the start and end of the perturbations indicated by the dotted lines and unperturbed standard deviation in gray) is shown as well as an perturbation-normalized plot of the difference (Δ) with the unperturbed strides (with half of the perturbations indicated by the dotted line).

In contrast with ACC, TA showed a clear response to DEC. An increase was found for TA (10/10 subjects), and occasionally for SO (2/10), while a decrease in muscle activity was seen in GM (10/10), GL (8/10) and sometimes for SO (3/10; Fig 5). The relative amount of EMG of all muscles was increased compared with unperturbed walking (all p<0.02) and increased with intensity (all p≤0.005). This difference in EMG was caused by an increased incidence of bursts in the EMG of TA (p<0.04) and of depressed EMG for the calf muscles (p = 0.004), the incidence of which increased with intensity (p = 0.007). Both the mechanical and EMG delay of the depression in calf muscles was independent of intensity, and between 29–53 ms for delay_mech_ and 149–197 ms for delay_EMG_, except for the increasing EMG delay of GM (p = 0.011). Muscle response strength increased with intensity for TA (*p* = 0.03), and remained similar (SO) or decreased with intensity for GM and GL (*p*≤0.05). Peak TA burst was positively related to MTV (*p* = 0.004). The level of co-contraction with TA was similar for GL and SO but 14% higher for GM during perturbed compared with unperturbed walking (*p* = 0.009), but did not change with intensity for any of the calf muscles.

The effect on the gait cycle was also opposite, with increasing stance phase duration and stride time as well as decreasing stride length (p<0.008). In contrast with ACC, step width was increased during the perturbed strides compared to the unperturbed strides and increasing with intensity (p = 0.015). An overview of the effects of ACC is given in Fig 4 and Table 3.

**Table 3:** Parameters DEC.

Parameters	UNPERTURBED		PERTURBED					
		Effect P	I1	I2	I3	I4	I5	Effect I
**Kinematics**								
Min angle (°)								
ankle	-0.7±0.3^3−5^	**0.004**	-0.9±0.9^3−5^	-1.3±1.3^3−5^	-2.1±1.1^5^	-2.8±0.9^5^	-3.4±1.2	**0.000**
knee	-0.8±0.2^2−5^	**0.001**	-0.9±0.9^3−5^	-1.4±0.5^4,5^	-2.3±1.0^-^	-2.8±1.3^-^	-3.6±2.2	**0.002**
**Muscle**								
Min MTL (x10^-3^)								
GM	-0.4±0.8^1−5^	**0.000**	-0.9±0.9^3−5^	-1.4±1.1^-^	-1.9±0.8^-^	-2.5±1.0^-^	-2.8±1.2	**0.000**
GL	-0.4±0.9^1−5^	**0.000**	-0.9±0.9^3−5^	-1.5±1.2^5^	-2.0±0.9^-^	-2.5±1.0^-^	-2.8±1.2	**0.000**
SO	-1.0±1.5^2−5^	**0.000**	-1.9±2.7^2−5^	-3.2±2.3^3−5^	-5.1±2.2^5^	-6.5±1.9^-^	-7.7±2.9	**0.000**
Max MTL (x10^-3^)								
TA	1.0±1.2^2−5^	**0.000**	1.9±2.3^2−5^	3.2±1.9^3−5^	5.0±1.7^5^	6.5±1.4^-^	7.5±2.2	**0.000**
Min MTV (x10^-3^)								
GM	-2.1±0.5^1−5^	**0.000**	-2.9±0.6^3−5^	-3.3±0.6^4,5^	-3.7±0.4^4,5^	-4.4±0.6^5^	-4.9±0.4	**0.000**
GL	-2.2±0.5^1−5^	**0.000**	-3.0±0.6^3-,5^	-3.3±0.7^3−5^	-3.8±0.4^4,5^	-4.5±0.6^5^	-5.0±0.4	**0.000**
SO	-3.6±0.8^2−5^	**0.000**	-5.0±1.8^2−5^	-6.1±1.4^3−5^	-7.2±1.9^4,5^	-9.0±2.0^5^	-9.9±2.5	**0.000**
Max MTV (x10^-3^)								
TA	3.3±0.6^1−5^	**0.000**	4.9±1.6^2−5^	6.0±1.2^4,5^	7.2±1.5^4,5^	8.8±1.4^-^	9.5±1.9	**0.000**
**EMG**								
RMS (%)								
GM	32±8^3−5^	**0.001**	32±9^2−5^	35±11^3−5^	39±10^5^	42±11^-^	45±10	**0.000**
GL	20±9^3−5^	**0.000**	19±8^3-,5^	21±9^4,5^	22±8^4,5^	24±8^-^	26±11	**0.000**
SO	21±8^1−5^	**0.005**	22±10^4^	23±8^4^	27±7^-^	31±10^-^	32±11	**0.005**
TA	8±4^5^	**0.014**	10±5^-^	18±19^4,5^	30±29^5^	36±32^-^	40±32	**0.009**
Bursts (%)								
GM	4±3	0.709	4±4	3±6	3±5	4±5	8±10	0.339
GL	4±3	0.080	2±3	2±3	2±3	5±6	2±4	0.373
SO	4±3	0.066	3±6^-^	5±7^-^	16±25^-^	24±29^-^	25±31	0.060
TA	6±3^3,5^	**0.037**	1±2^3,5^	10±12^-^	9±10^-^	15±16^-^	17±10	**0.006**
Delay_mech,burst_ (ms)								
SO						38±30	17±13	0.548
Delay_EMG,burst_ (ms)								
SO						144±70	161±44	0.243
Depression (%)								
GM	4±4^3−5^	**0.000**	5±8^2−5^	23±22^4,5^	45±35^4,5^	62±32^-^	69±28	**0.000**
GL	1±1^4,5^	**0.006**	2±4^4,5^	6±6^4,5^	18±17^-^	28±25^-^	29±27	**0.004**
SO	2±2^3−5^	**0.003**	1±3^3−5^	7±9^-^	19±18^-^	24±23^-^	26±24	**0.001**
TA	0±1	0.078	1±3	0±0	0±0	0±0	0±0	0.083
Delay_mech,depres_ (ms)								
GM				45±25	48±25	53±36	49±28	0.570
GL						31±28	39±22	0.511
SO						29±30	34±30	0.550
Delay_EMG,depres_ (ms)								
GM				149±45	164±16	165±49	173±40	**0.011**
GL						192±33	192±30	0.998
SO						187±63	197±77	0.477
Strength (x10^3)^								
GM	-56±16^1−5^	**0.000**	-28±13^4,5^	-25±17^4,5^	-19±14^-^	-17±11^-^	-19±13	**0.001**
GL	-34±14^1,3–5^	**0.001**	-17±10^5^	-18±9^-^	-12±4^-^	-12±4^-^	-11±9	**0.048**
SO	-21±10^1,2^	**0.010**	-14±5	-10±3	-11±5	-11±5	-10±6	0.288
TA	7±4	0.088	6±7^5^	13±19^-^	16±19^-^	17±17^-^	16±14	**0.026**
CCI (%)								
GM	0±0^3^	**0.009**	0±0^-^	0±0^-^	0±0^-^	0±0^-^	0±0	0.092
GL	1±0	0.458	1±0	1±0	1±0	1±0	1±0	0.076
SO	1±0	0.335	1±0	1±0	1±0	1±0	1±0	0.298
**Spatiotemporal**								
Stride length (m)	1.33±0.04^3,5^	**0.003**	1.32±0.05^-^	1.31±0.05^-^	1.31±0.06^5^	1.30±0.06^-^	1.29±0.06	**0.007**
Stride time (s)	1.11±0.04^3−5^	**0.000**	1.11±0.04^3−5^	1.12±0.04^3−5^	1.13±0.05^5^	1.14±0.05^-^	1.14±0.05	**0.000**
Stance phase (%)	65.8±0.7^1−5^	**0.000**	66.2±0.7^2−5^	66.6±0.5^4,5^	66.9±0.8^4,5^	67.2±0.8^-^	67.5±0.8	**0.000**
Step width (cm)	10.6±2.6^3,4^	**0.001**	10.6±2.9^-^	10.9±2.5^-^	11.2±2.2^-^	11.3±2.6^-^	11.3±2.7	**0.015**

With max maximum, min minimum, RMS root-mean-square, CCI co-contraction index, Strength the response strength and I Intensity. Grand means and standard deviations are given. Mechanical and EMG delays are only shown if sufficient group data was available. Effect P shows the p-values of the significant difference between unperturbed versus all perturbed and Effect I shows the p-values of the effect of intensity. Results of the post-hoc tests are indicated with in superscript the higher intensities that differ from a specific trial (p<0.01).

## Discussion

This paper gives a comprehensive overview on the effects of acceleration and deceleration perturbations with varying intensities on reflex activity of all major lower leg muscles during the stance phase of unconstraint treadmill walking in healthy subjects. It was found that the ACC perturbations resulted in small dorsiflexion of the ankle, causing an elongation of the calf muscles and subsequently bursts in their muscle activity. The antagonist TA was shortened without showing a muscle response. The DEC perturbations caused a small plantar flexion of the ankle and shortening of the calf muscles, resulting in a depression in their activity. The lengthened TA showed bursts in the muscle activity.

### Stretch reflexes

Our results met all five conditions we formulated to determine if stretch reflexes occurred, and hence we can conclude that the perturbations were able to evoke stretch reflexes. Stretched muscles showed an increase in muscle activity, which was not related to co-contraction but to the incidence of bursts in the EMG. These bursts occurred after a reasonable delay of 163–191 ms. The electrophysiological delays of the bursts in the calf muscles (delay_EMG,burst_) were in the order of 144 to 191 ms. This is outside the time window of 70–120 ms expected for long-latency reflexes in the lower leg muscles and a bit longer than found in previous studies [12–14;18;19]. However, the MTV-threshold was chosen rather conservatively (one standard deviation), so that for almost all cases with bursts in the EMG also an MTV threshold was found. This resulted in a probable overestimation of the true electrophysiological time delays. Besides the unknown stretch velocity threshold, the filter frequency of the EMG and definition of a burst in the EMG, i.e. the threshold and minimal duration, also had an effect on the measured time delay. Our low-pass filter cut-off frequency was comparable to earlier studies (30–40 Hz), however, the definition of a burst differed from previous studies. They varied from visual inspection to absolute EMG thresholds (of 10 to 25 uV or 2 standard deviations) from a baseline within a strict time window (e.g. 22–100 or 20–60 ms after start of the perturbation) [11;12;19;20;22]. A more limited time window and less strict values for the minimal peak duration and minimal peak amplitude might have resulted in shorter time delays in our study compared with these studies.

Since the mechanical delay describes the time from start of belt perturbation until a notable change in MTV, it was expected to decrease with intensity, while the electrophysiological delay was expected to remain constant. The latter was indeed the case, but the mechanical delays did not decrease with intensity. It might well be that these time differences between intensities were too small to be detected, while there was no obvious criterion to use for the mechanical threshold. The necessity of estimating a mechanical delay resulted from the relatively long duration (up to 336 ms) of the perturbations used in this study, which was due to constraints of the current system. The relative long perturbation duration limited the discrimination between the stretch reflexes and ongoing perturbation effects. The duration was longer than those of the accelerations used by Berger and colleagues of 200 ms [25] and considerably longer than the rapid rotations applied by strapped devices (18–100 ms) [13;14;19;21]. The difficulty with discriminating between the mechanical and electrophysiological delay also hindered the distinction between short and long latency reflexes. Occasionally, delays were measured that fell within the range for short latencies (up to 40 ms [18]), but most bursts had a longer time delay. This is consistent with the findings of Berger and colleagues in healthy subjects, despite the higher intensity of their perturbations [11;25]. Since spastic patients have been shown to have exaggerated short latency reflexes [20–22], the discrimination between short and long latencies becomes more important and feasible when measuring reflexes in these patients.

The amplitude of the EMG bursts was positively related to muscle stretch velocity, demonstrating the velocity-dependence of the muscle responses. In addition, since muscle velocity and length changes were found to be correlated, the reflexes also showed a significant positive correlation to muscle length change (all muscles *p*≤0.001). It should be noted that the musculoskeletal modeling that was used to estimate the muscle-tendon unit length and velocity changes from the ankle angle could not discriminate between the effect on muscle fascicles and the relatively long Achilles tendon. However, soleus muscle fascicle changes have been shown to follow the changes in ankle angle induced by a mechanical joint [35]. Future studies would benefit from including ultrasound measurement to validate the muscle fascicle lengthening due to the perturbations.

### Unloading response

Since the modulation of the functional contribution of reflexes to muscle activity has been shown to be altered in patients during muscle unloading [22;24], the unloading response was also examined in this study. By decreasing the muscle length, both the position and velocity feedback reduced [19;36]. This resulted in a depression in the EMG of all calf muscles, the incidence of which increased with intensity. At the highest intensity, the incidence was up to 69% in GM, but to less than 30% in GL and SO. This is consistent with the more pronounced reduction in response strength in GM compared with GL and SO. Interestingly, GM also showed an increase in the level of co-contraction with TA compared with unperturbed strides. This could be caused by the increasing incidence of a depression, which reduces the difference in GM and TA muscle activity, together with the occurrence of a late rebound response after the depression period, as has also been found during electrical tibial stimulation [37]. The time delay after which the depression occurred was similar to the delay of bursts in stretched muscles, as was expected since the same feedback pathways are involved.

During the ACC perturbations, however, TA did not show any reductions in the EMG, corresponding with previous SO stretching studies [14;19;21]. This could be related to the already low activity in TA during the later stance phase, or to small activations occurring simultaneously with elongation of the gastrocnemius as has been reported previously [11]. These activations would have been too small to be detected as a bursts, but it would explain the lack of a reduction in co-contraction compared with unperturbed gait as is seen for the GL and SO. It should be noted that the effect on muscle activity cannot be attributed to a reduction in a specific afferent feedback pathway, since not only the length and velocity feedback are reduced, but also the force sensitive feedback [19;36].

### Perturbation intensities

The highest intensity reached a belt speed difference of 0.42 ms^-1^, which is lower than the anticipated 0.5 ms^-1^. This could be due to the leveling off of the maximum acceleration to 0.29 ms^-2^ for the higher intensities (Fig 2), and explain that lack of significant differences in muscle response between middle and high intensities. There was also a relatively large difference in maximum velocity between trials of the same intensity within a subject, possibly reflecting interactions between the treadmill belt and subject during the perturbation [38]. Even so, the maximum perturbation resulted in changes in ankle angle of up to an individual average of 4.9° for ACC and 5.7° for DEC for individuals, with most parameters showing a linearly increase with intensity. The studies by Berger and colleagues used perturbations of considerably higher intensities, with belt speed differences of up to 5 km/h for ACC (19.8 ms^-2^) and 2.5 km/h for DEC (11.6 ms^-2^). They reached much higher total ankle angular velocities (up to 250–300°s^-1^)[25] compared to our total angular velocities, with 60–100°s^-1^ muscle lengthening during an unperturbed gait cycle and up to 50°s^-1^ induced by our perturbations. Unfortunately, they did not report the change in ankle angle. The actuated joints and pneumatic devices used in other studies also reached higher peak angular velocities (40–500°s^-1^) [13;14;19;21], which is more in the range of manual clinical spasticity tests of the calf muscles (180–720°s^-1^) [39]. Even though these studies applied higher intensities, only slightly larger ankle rotations were reached compared with our results (3–9°) [13;14;19;21].

The optimal perturbation intensities are a compromise, they should be low enough to limit major disturbance of the gait cycle and high enough to consistently elicit stretch reflexes. Our perturbations intensities were lower than used in previous studies [13;14;19;21;25], but reasonable incidence of reflexes were measured at the highest intensity for the calf muscles. However, the average incidence of stretch reflexes (bursts in EMG) did not reach a full 100%, but stayed at 40 to 60%. This can be caused by the rather strict definition of a burst (exceeding a high threshold and minimal time window) used in this study. It is also an indication that while in some subjects the highest intensity always elicited a burst in EMG, the intensities should have been higher to consistently evoke a response in other subjects. Unfortunately, response rates were not consistently reported in other studies, while comparison is hampered by different definitions of stretch reflexes. Another important factor to consistently measure stretch reflexes is to apply the perturbations at the same moment during the gait cycle, since stretch reflexes have been shown to be modulated during a stride [18;21]. The deviation in start of the perturbation was due to inherent technical limitations of the equipment, variation in stride duration and possibly to a small difference in detection of initial contact during the and offline analysis. However, the variation was limited to only 2% of the gait cycle.

The requirement that perturbations only caused limited gait disturbance is not only important to measure representative gait, but also for the method to be suitable for clinical application. The perturbations used in this study did seem to have a limited effect on the gait pattern. The highest intensities resulted in less than 4° knee flexion or extension. In addition, stride length changed with less than 4%, stride time and stance phase changed less than 3.5%, while part of these changes inherent of the perturbation. Step width increased by 6.1% during DEC. This could be an attempt to increase the base of support in the medio-lateral direction, after it decreased in anterior-posterior direction due to the deceleration [40]. During ACC, step width remained unchanged indicating that subjects were not disturbed by the perturbations, although the effect might be larger in patients especially for those with stability problems. In addition, the unperturbed strides that were used for comparison, were sampled from the same trials. While this limited the effects of time, fatigue, and previous perturbations, it cannot be excluded that anticipation to perturbations affected the strides [41]. Future studies should examine the application of shorter perturbations, to see if the incidence of elicited stretch reflexes increases, if the mechanical delay can be neglected and if short latency reflexes can be discriminated easier, while the gait pattern remains undisturbed. In addition, the effect of the perturbations on the upper body and a subject’s stability as well as the occurrence of any compensational strategies should be further examined.

### Clinical application

Our findings indicate that applying treadmill perturbations of different intensities is a potential useful method to define exaggerated reflexes during gait in patients. The response strength measure shows that muscle activity increased relative to an increasing stretch velocity for GM, GL and TA, with a similar trend for SO. This measure is derived from dynamic spasticity, which has been used to demonstrate an exaggerated reflex response to increasing stretch velocity during the swing phase of children with CP relative to typically developing children [8]. The response strength thus shows potential to quantify hyperreflexia in patients with spasticity measured with treadmill perturbations. It should be noted that the relation between muscle response and stretch velocity is a complex, non-linear function that depends on force, muscle length, and gait phase. Clinical implementation would benefit from a clinically meaningful and robust quantification of reflex responses that takes this complex relation into account. Next to ACC measurements, DEC measurements seem also relevant, not only to examine the reflexes in TA, but also because previous studies have shown that the modulation of the reduction in muscle activity of a shortened muscle is altered [22]. It should be noted, however, that the effect of the perturbations as shown for the healthy subjects cannot be automatically generalized to all spastic patients, but most likely depends on the gait pattern, especially the angle at mid stance, of a specific patients. Previous studies have shown that similar perturbations could evoke stretch reflexes in a limited set of subject with mild spasticity [25;42]. Future studies should further elaborate on the effect of the perturbations on patients, for instance those that exhibit an abnormal gait pattern, such as toe walking or crouch gait. The current study provided normative data that enables evaluation of patients against the response of normal subjects.

The disadvantage of treadmill perturbations is that the perturbations can only be applied during the stance phase of gait. This way, potential exaggerated reflexes cannot be examined during the swing phase of gait. In addition, the perturbations might not be strong enough to elicit stretch reflexes in the major thigh muscles, although a recent study has shown increased muscle responses in the biceps femoris muscle after comparable treadmill perturbations [43]. On the other hand, treadmill perturbations do not require any strapping, which hampers normal movement especially in little children, nor does it require longer familiarizing time to actuated devices. Moreover, it potentially allows for measurement while the patients can wear their normal orthopedic shoes or orthoses.

As a next step, the effect of the perturbations should be examined in spastic patient groups and compared with conventional clinical tests. Before it can be applied, however, it should be determined if fewer intensities can be used to reduce the measurement time and if similar but shorter perturbations can be applied while not affecting the gait stability. Furthermore, the modulation of mechanical properties of the ankle, such as its stiffness, could be incorporated, by measurement and analysis of ankle moment [44], as well as the measurement of dynamic gait stability, in order to quantify the effect of the perturbations on the stability [45].

### Conclusion

Our findings show that belt speed perturbations during walking on a treadmill can evoke muscle reflexes in the calf muscles and TA in healthy subjects. A clear unloading effect was found for the calf muscles after deceleration perturbations. Although the effect on the ankle angle was only a few degrees, the perturbations caused a significant increase in elongation and stretch velocity of all muscles, followed by clear bursts or depression of muscle activity. The perturbations only slightly affected the gait pattern, indicating that the gross walking pattern was retained. These findings establish a basis for implementation of belt speed perturbations to functionally measure spasticity during treadmill-based clinical gait analysis by providing normative data to compare responses from spastic patients with.

## Supporting Information

S1 Dataset The dataset contains relevant outcome parameters for individual subjects. (XLSX)

S1 Fig The recovery during strides after the perturbation of the highest intensity. (TIF)
