# Supplementary figures and images for: Can Treadmill Perturbations Evoke Stretch Reflexes in the Calf Muscles?
Source: PLoS One. 2015 Dec 15;10(12):e0144815. doi: 10.1371/journal.pone.0144815 (PMC4682928; doi:10.1371/journal.pone.0144815)

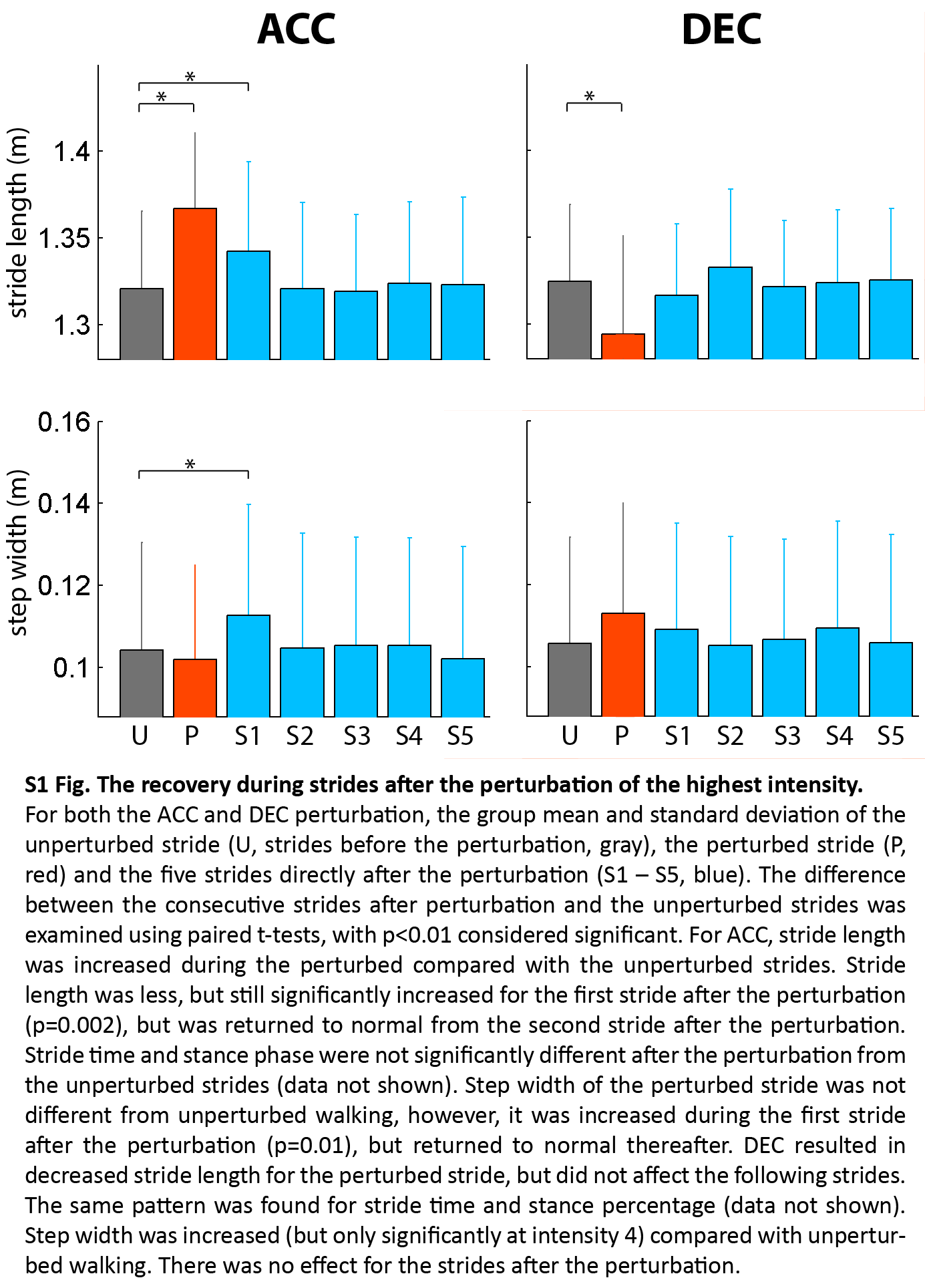

Supplement: S1 Fig — (TIF) [file pone.0144815.s002.tif]
